# Development of a multiyear pediatric antibiogram in Georgia identifies antibiotic resistance trends

**DOI:** 10.1017/ash.2023.256

**Published:** 2023-09-29

**Authors:** Matt Linam, Madeleine Goldstein, Robert Jerris, Mark Gonzalez

## Abstract

**Background:** Antibiograms are used to monitor antibiotic resistance trends and help guide empiric antibiotic treatment. Community pediatricians may not have access to or be comfortable using children’s hospital antibiograms. Creating and disseminating a statewide pediatric antibiogram can help inform antibiotic stewardship efforts.

**Objective:** To develop a pediatric-specific antibiogram for the state of Georgia. **Methods:** Annual pediatric antibiograms for the 5 children’s hospitals in Georgia from 2014 through 2021 were collected. All sites complied with the Clinical and Laboratory Standards Institute guidelines for antibiomicrobic breakpoints and antibiogram development. Antibiogram data were combined, and the most common bacteria were selected to incorporate into the statewide antibiogram: *Staphylococcus aureus*, *Streptococcus pneumoniae*, *Enterococcus faecalis*, *Escherichia coli*, *Klebsiella pneumoniae*, *Enterobacter cloacae* complex, and *Pseudomonas aeruginosa*. Antibiogram data were reported as percentage susceptible and total number of isolates. Interhospital susceptibility differences were compared for methicillin-susceptible *S. aureus* (MSSA), methicillin-resistant *S. aureus* (MRSA), *E. coli*, and *K. pneumoniae* from 2018 through 2021. *P* < .05 was considered significant. The combined antibiogram data from 2014 through 2021 were used to show antibiotic susceptibility trends over time. **Results:** The 2021 antibiogram is shown in the Table. For MSSA and MRSA, clindamycin susceptibility was 80% and 85%, respectively. *K. pneumoniae* susceptibility to amoxicillin-clavulanate was 91%. For *E. coli*, using urine-specific breakpoints, susceptibility to cefazolin was 89%. A few statistically significant differences in antibiotic susceptibility were detected between hospitals, but most were unlikely to be clinically relevant (all susceptibilities ≥90% or < 80%). A notable exception was trimethoprim-sulfamethoxazole susceptibility for *K. pneumoniae*, which ranged from 74% to 98% in 2020 and from 74% to 86% in 2021. From 2014 to 2021, the percentage of MRSA decreased from 49% to 34%. Over the 8 years, susceptibility to ceftriaxone for *E. coli* ranged from 93% to 95% and from 90% to 95% for *K. pneumoniae*. Susceptibility to meropenem for *E. coli* and *K. pneumoniae* ranged from 99% to 100%. **Conclusions:** Antibiotic susceptibility for pediatric bacterial isolates in Georgia remained stable over time and supported the narrow-spectrum empiric antibiotic treatment recommended in national evidence-based guidelines for skin and soft-tissue infections, community-acquired pneumonia, and uncomplicated urinary tract infections. MRSA rates decreased over time and multidrug-resistant gram-negative bacilli were uncommon and remained stable.

**Figure:**
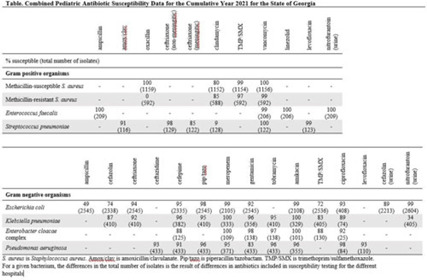

**Disclosures:** None

